# Supplementary material for: Pollution-Free and Highly Sensitive Lactate Detection in Cell Culture Based on a Microfluidic Chip
Source: Micromachines (Basel). 2023 Mar 30;14(4):770. doi: 10.3390/mi14040770 (PMC10146269; doi:10.3390/mi14040770)
Supplement: Supplementary file 1 [file micromachines-14-00770-s001.zip › micromachines-2284788-supplementary.pdf]

Electronic Supplementary Information

The parameters in particle tracking simulation are optimized, including the time interval of particle release (Figure S1), particle number (Figure S2) and grid number (Figure S3). In these three figures, the fluctuation of the results gradually decreases, and the curve gradually becomes smooth. Therefore, the final selected time interval of particle release, particle number and grid number are 0.5s, 250k, and 9.6 million respectively.

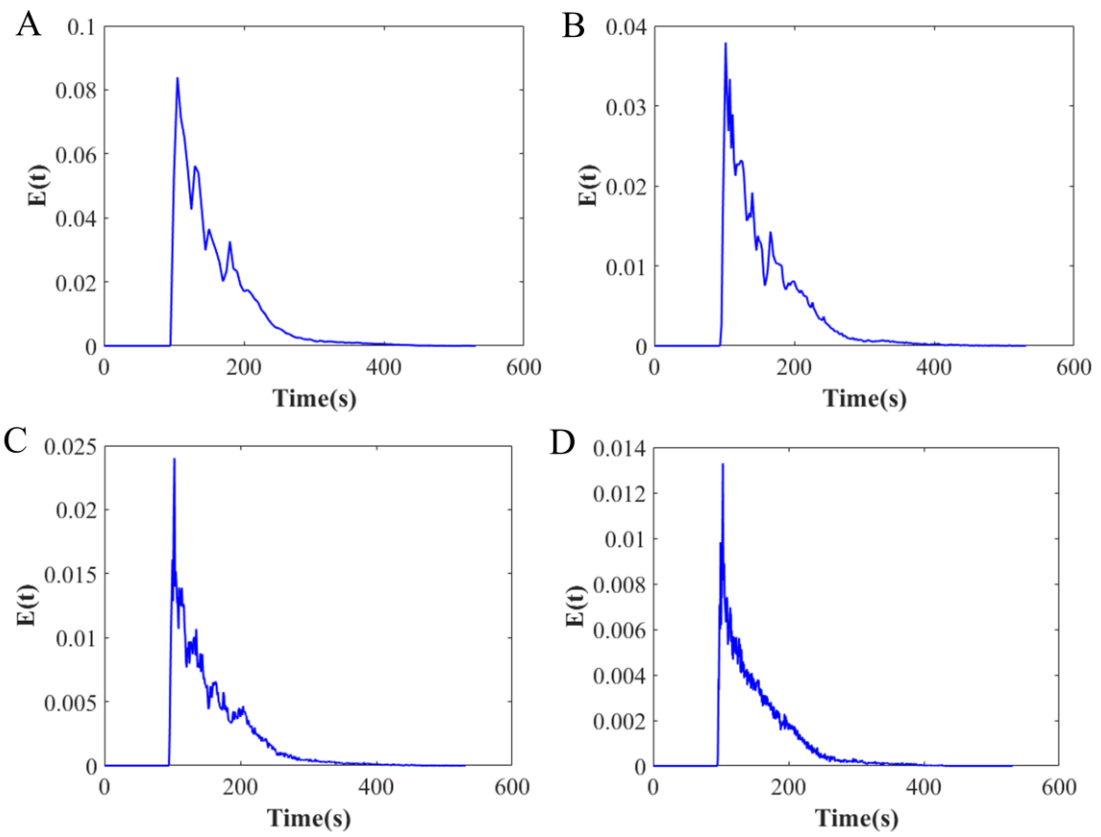

Figure S1. The influence of particle release time interval on simulation results. The time interval from A to D is 5s, 2s, 1s and 0.5s respectively.

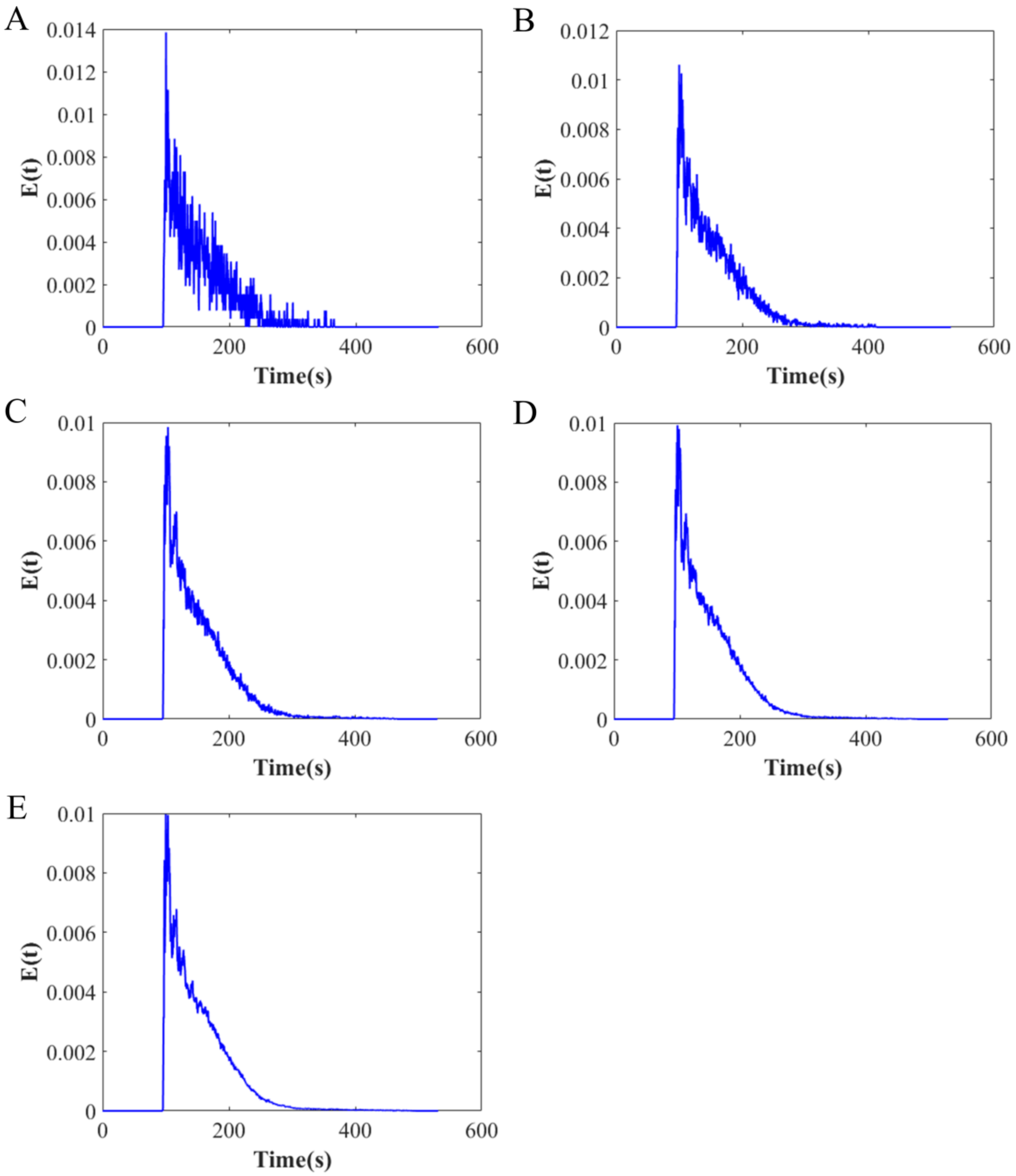

Figure S2. The influence of particle number on simulation results. The particle number from A to E is 2600, 22k, 90k, 250k and 560k respectively.

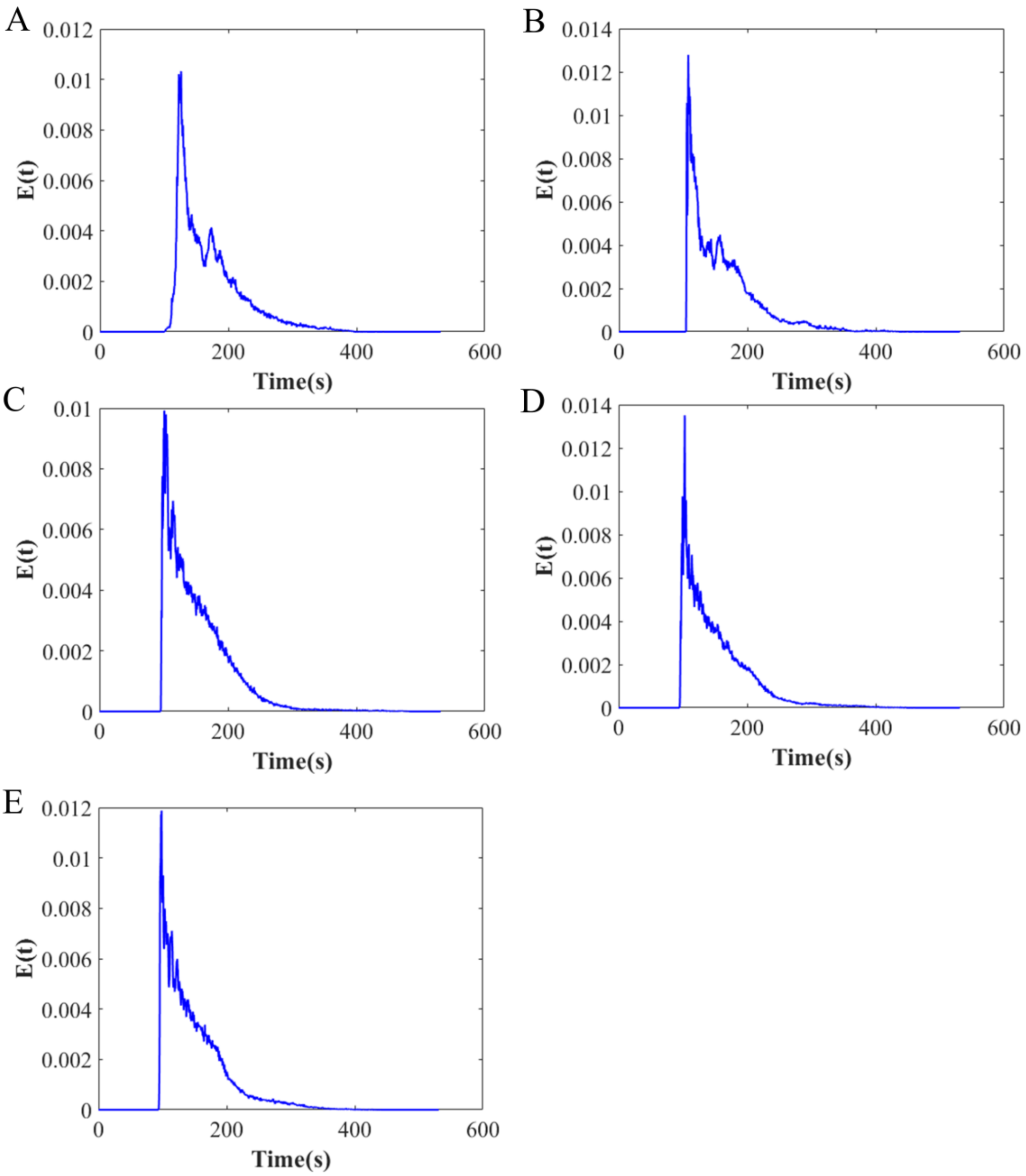

Figure S3. The influence of grid number on simulation results. The grid number from A to E is 0.3 million, 0.8 million, 3.7 million, 4.6 million and 9.6 million respectively.
